# 

**Published:** 1889-06

**Authors:** 


### INDEX TO VOLUME LVIII.

### A.

PAGE
Abortion in the Third Month, A Case of. Elbert Wing ................ 196
Abscesses, Opening Deep Peri-Rectal, by Perineal Incision ......... 215
Anæmia, Salt Infusion in Acute .... 85
Anæsthesia, Methyl Chloride for Local ............................. 151
Antipyrin in Chorea ................ 20

### B.

Bacillus Tuberculosis, The Detection of the. Frank Billings ......... 131
Books Received ........... 121, 192, 377
Book Reviews and Notices, 117, 191, 252 310, 374 .....................
Bread, Alum in .................... 140
Buboes, Surgical Treatment of Suppurative Venereal ................ 9
Butyl-chloral in Trigeminal Neuralgias ............................ 87

### C.

Camphor, and Ammonium Salts as Stimulants ..................... 87
Cannabin in Graves Disease ........ 217
Cancerous Diseases, Curability of ... 7
Charity Institutions of Paris ....... 274
Chloroform, Benzoated ............ 216
Chorea, Antipyrin in .............. 20
Clinical Reports .................. 93
Cocaine Habit in Mother and Child .. 338
Cocaine Habit in Diseases of the Throat and Nose ................ 337
Codeine as a Substitute for Morphine 14
Confirmed Catheter Life, Treatment of by a Permanent Perinæal Opening ............................ 263
Constipation, Glycerine in .......... 88
Cornea, Treatment of Serpiginous Ulcers of the .................. 217
Cork as a Compress in Hæmorrhages . 214
"Cremator" Cremated ............ 276
Creosote in Pulmonary Phthisis ..... 15
PAGE
Crude Sulpho-Carbolic Acid as a Means of Disinfection ........... 216

### D.

Diabetic Coma, Nature and Treatment of ........................ 141
Diabetes Mellitus, The Relative Value of Opium, Morphine and Codeine in 206
Diabetes Mellitus, Opiates in ....... 142
Diphtheria, Antiseptic Pastiles in ... 87
Dyspepsia as Reflected in the Upper Air Passages .................. 268
Dyspnœa, Dyspeptic ............... 10

### E.

Ear, Hæmatoma of the, in Foot-ball Players ........................ 13
Ear, Treatment of Furuncle of ..... 14
Eczema, An Anomalous Form of .... 138
Eczema, Treatment of ............. 87
Eclampsia, Citrate of Caffeine in ... 154
Ectropium, Transplantation of Skin for the Cure of. H. Gradle .... 133
Electricity, The Clinical Uses of. D. R. Brower .................... 1
Ergot in Heart Disease ............ 86
Erysipelas, Curative .............. 8
Epicondyles, Fracture of ........... 85
Epicystic Surgical Fistula for Cystroscopic Exploration: Intra-vesical Treatment and Drainage. John D. S. Davis ......................... 257
Epistaxis, Oleum Origanum in ...... 86
Ethylene Chloride, Action of Inhalations of, in the Eye ..............
Expectorant Mixture .............. 88
Experiments to Determine the Antiseptic value of Boric Acid. E. Andrews ......................... 129
Extracts and Abstracts, 5, 73, 134, 201 262, 327 ........................
Eye, Action of Inhalations of Ethelene Chloride on the ............ 217
Eye, The Tabetic ................. 271
Eyes, Congenital Lateral Deviation of the ......................... 84

F.

PAGE

Faradism in Hysteria and Epilepsy.. 8
Fibroid Tumors of the Uterus: Locking, Retroversion and Strangulation of, in the Pelvic Excavation...... 77
Fibroid Tumors of the Uterus, Medical Treatement of............... 73

G.

Gastric Juice, The, in Febrile Diseases.......................... 15
Germany, Overcrowding of the Medical Profession in............... 276
Gluten as an Article of Diet........ 156
Glycosuria, Jambul in............. 20
Goitre, Exophthalmic, Electricity in.. 85
Graves Disease, Cannabin in........ 217
Guy's Hospital Lying, in Charity.... 208

H.

Headaches, Chronic, of Functional Origin.......................... 152
Heart Disease, Ergot in............ 87
Helleborin. as a Local Anæsthetic... 87
Hernia, The Radical Cure of Inguinal 272
Hydatids, Injection of Ox-Gall in... 87
Hydroxylamin in Skin Diseases..... 144
Hysteria and Epilepsy, Faradism in.. 8

I.

Inhalations, The Value of, in the Treatment of Lung Disease........... 153
Intussusception in Infants—Laparotomy and Exsection. A. B. Strong 65
Iso-Nitroso—Antipyrin............. 217

J.

Japan, Medicine in................ 276

K.

Knee, Arthrectomy versus Excision of the.......................... 179

L.

Larynx, Illumination of, by Transmitted Light..................
Leprosy Cured by Unna's Method... 137
Lead-Poisoning from Service Pipes in Relation to Sterility and Abortion............................ 149
Leprosy, Nerve Stretching in....... 81
Litholapaxy, Two Cases in which L. was Impossible. Edmund Andrews 262
Lung Disease, The Value of Inhalations in the Treatment of........ 153

M.

PAGE

Mammary Inflammation, Elastic Pressure in......................... 18
Mechanical Treatment of Hip-joint Disease. Dr. Wallace Blanchard. 321
Mental Depression and the Excretion of Uric Acid................. 153
Menthol and Iodoform in Surgery... 86
Methyl Chloride for Local Anæsthesia.......................... 151
Miscellany...........122, 254, 314, 378
Myrtol as a Respiratory Disinfectant 86

N.

Nasal Affections, The Treatment of.. 154
Navy—Medical Department of the U. S. Navy...................... 11
Neuralgia, The Differential Diagnosis of. D. R. Brower.............. 198
Neuralgia, The Etiology of. H. M. Lyman......................... 193
Neuralgias, Trigeminal Buttyl-Chloral in........................... 87
New Drugs, Notes on the Value of Some......................... 201
Nursing Question, The............. 275
Nursing Mothers, What Medicines May be Given................... 88

O.

Obesity, Water in................. 20
Obstetrics, Bichloride of Mercury in.. 86

P.

Pamphlets Received...........122, 254
Paris, The Charity Institutions of ... 274
Patella—Subcutaneous Ligature in Fracture of..................... 148
Pericarditis, Aspiration and Drainage in Purulent................. 12
Pernicious Anæmia, A case of. F. J. Hodges....................... 200
Phenacetin....................... 145
Pneumonia, The Contagiousness of.. 156
Potatoes, Dried................... 276
Pott's Disease of the Spine, A Practical Point in the Treatment of..... 213
Prostate, Radical Cure of Hypertrophy of...................... 216
Puerperal Eclampsia, Morphine in... 88
Puerperal Fever, Treatment of. C. W. Earle...................... 67
Puerperal Fever, The Bacteriology of 143
Puerperal Insanity of Septic Origin.. 17
Puerperal Septicæmia, Treatment of. 88
Puerperal Uterus, How to Irrigate the 135

S.

PAGE

Sacro-Iliac Joint, Tuberculosis of.... 215
Salivary Calculus, A Case of, with Removal........................ 337
Shock, Surgical.................... 144
Skin Diseases, Hydroxylamin in..... 144
Skin Transplantation for the Cure of Ectropium. H. Gradle.......... 133

SOCIETY REPORTS.

Academie de Medecine........... 308
Allegheny County Medical Society ......................60, 224, 280
British Gynecological Society..361, 373
Chicago Medical Society.....26, 45, 97 161, 169, 233, 240, 241, 292, 295
Chicago Pathological Society...30, 243
Clinical Society of London....... 368
Gynecological Society of Chicago.. 34
Gynecological and Obstetrical Society of Baltimore 99, 101, 108, 183, 349
Harveian Society of London...250, 296
Hunterian Society of London..... 373
Illinois State Medical Society..... 340
Odontological Society of Great Britain.......................... 307
Medical Society of Berlin........ 309
New York Academy of Medicine 286, 359
Royal Academy of Medicine...... 301
The Medical Society of London... 365
The National Association of Railway Surgeons................ 355

Spine, Bavarian Plaster Jacket for Fractured...................... 13
Spontaneous Strangulation of the Limbs ........................ 11
Strychnia as an Antidote in Narcotic Poisoning..................... 18
Summer Diarrhœa, Prevention of, in Children....................... 139
Syphilis, Treatment of............. 19

T.

Tabes Dorsalis, Treatment of........ 84
Tape-worm in an Infant on Raw Meat Diet.......................... 141
Terpene as an Expectorant......... 88
Testis—Transplantation from Perinæum......................... 134
Tetanus, The Etiology of.......... 275
Thymol in the Treatment of Tuberculosis .......................... 155
Thymus Gland, Extirpation of the... 155
Torsion of Arteries for the Arrest of Hæmorrhage. Dr. J. B. Murdoch. 325
Tuberculosis from Contagion....... 156
Tuberculosis of the Sacro-Iliac Joint 215

PAGE

Tuberculosis Pulmonum, Creosote in 15
Tuberculosis—Treatment of T. Pulmonum.......................... 83
Typhlitis, Treatment of............ 20
Typhoid Fever, Beta-Naphthol in... 217
Typhoid Fever, Germicides in...... 146

U.

Uterus, Fibroid Tumors of.......73, 79
Uterus, How to Irrigate the Puerperal 135
Uric Acid, Quantitative Estimation of 19
Uric Acid—The Excretion of, and Mental Depression.............. 153

V.

Vaginismus, Treatment of...........155

Y.

Yeast in Infectious Diseases........ 20

CONTRIBUTORS TO THIS VOLUME.

| | |
|---|---|
| EDMUND ANDREWS. | HENRY GRADLE. |
| FRANK BILLINGS. | F. J. HODGES. |
| WALLACE BLANCHARD. | H. M. LYMAN. |
| D. R. BROWER. | J. B. MURDOCH. |
| JOHN D. S. DAVIS. | A. B. STRONG. |
| C. W. EARLE. | ELBERT WING. |

Editorial.

A Decision on Medical Practice Acts 93
Bad Water, Filth and Typhoid Fever ......................... 92
Brain Weight and Intelligence.... 25
Cancer by Skin Grafts........... 92
Emergency Appliances on Railways 21
Influence of Salicylic Acid on the Excretion of Uric Acid......... 23
New Methods of Treating Loco-Motor Ataxia................. 278
Pharmacology and Therapeutics in 1888........................ 89
Physicians as Experts and Counsel 158
Pulmonary Tuberculosis and its Treatment by Hot Air......... 277
Pyrodine, a New Antiseptic....... 21
The Acquired Ileo-Femoral Articulation ........................ 22
The Apex-Beat in Children....... 93
The Effects of Tight Lacing...... 223
The Illinois State Board of Health. 280
The Journal of the American Medical Association................ 25
The State Board of Health Report. 157
The Treatment of Diabetes....... 218
Thiersch's Method of Skin Grafting 221
Toxic Effect of Antipyrin......... 339
Water Supply and Stream Pollution in Illinois..................... 220

### BOOKS RECEIVED.

The Operations of Surgery. By W. H. A. Jacobson, F.R.C.S.

Saint Bartholomew's Hospital Reports. Vol. XXXIX, 1888.

Reference Hand-book of the Medical Sciences. Vol. VII.

Transactions of the American Surgical Association. Vol. VI. J. Ewing Mears, Editor.

Rectal and Anal Surgery. By E. and E. W. Andrews.

Intestinal Surgery. By N. Senn, M.D., Ph.D.

A Compendium of Dentistry. By Julius Parreidt. Translated by Louis Ottofy, D.D.S.

Clinical Lectures on Diseases of the Urinary Organs. By Sir Henry Thompson.

Text-book of Medical Jurisprudence and Toxicology. By John J. Reese, M.D.

Hand-book of the Diagnosis and Treatment of Skin Diseases. By A. Van Harlingen, M.D.

Œuvres Complètes. Vol. V. de J. M. Charcot.

Transactions of the American Ophthalmological Society. Twenty-fourth Annual Meeting. 1888.

Hand-book of Physiology. By W. M. Baker, and V. D. Harris.

The Psychic Life of Micro-Organisms. By Alfred Binet. Translated by Thomas McCormack.

Prompt Aid to the Injured. By Alvah H. Doty, M.D.

A Hand-hook of Therapeutics. By Sidney Ringer, M.D.

Massotherapeutics, or Massage as a Mode of Treatment. By William Murrell, M.D., F.R.C.P.

Surgical Bacteriology. By Nicholas Senn, M.D.

Lectures on the Errors of Refraction and their Correction with Glasses. By Francis Valk, M.D.

The Insane in Foreign Countries. By William P. Letchworth.

Morrow's Atlas of Venereal and Skin Diseases. Fasiculi 10—11—12.

The Student's Text-Book of the Practice of Medicine. By Angel Money, M.D.

A Clinical Atlas of Venereal and Skin Diseases. Parts V. and VI. By R. W. Taylor, A.M., M.D.

An Elementary Treatise on Human Anatomy. By Joseph Leidy, M.D., LL.D.

Diseases of the Ear: Their Prevention and Cure. By C. H. Burnett, A.M., M.D.

Transactions of the Forty-Third Meeting of the Ohio State Medical Society, 1888.

A Manual of Diseases of the Ear. By Albert H. Buck, M.D.

Diphtheria: Its Nature and Treatment. By C. E. Billington, M.D.; and Intubation in Croup and other Acute and Chronic Forms of Stenosis of the Larynx. By Joseph O'Dwyer, M.D.

The Diagnosis and Treatment of Extra-Uterine Pregnancy. By John Strahan, M.D., etc.

Diabetes: Its Cause and Permanent Cure. Emil Schnée, M.D.

Physiological Notes on Primary Education and the Study of Language. By Mary Putnam Jacobi.

Examination of Water for Sanitary and Technical Purposes. Henry Lefferman, M.D.,Ph. D., and W[illegible]iam Beam, M.D.

The Bacteria in Asiatic Cholera. By E. Klein, M.D., F.R.S.

Extra-Uterine Pregnancy. A Discussion.

On the Binocular Metamorphosia Produced by Correcting Glasses. By J. A. Lippincott, M.D.

The Galvanic Treatment of Fibro-Myomata. By A. H. Buckmaster, M.D.

Tuberculosis of the Sacro-Iliac Joint. By Weller VanHook, M.D.

Electricity in Diseases of Women. By G. Betton Massey, M.D.

Affections of the Mediastinum. By H. A. Hare, B.Sc., M.D.

Hand-book of the Diagnosis and Treatment of Diseases of the Throat and Nose and Naso-Pharynx. By Carl Seiler, M.D.

The Year-Book of Treatment for 1889.

Sixteenth Annual Report of the Secretary of the State Board of Health of Michigan, 1888.

Second Biennial Report of the North Carolina Board of Health, 1889.

### MISCELLANY.

COLLABORATORS:

CHICAGO:

Professor E. ANDREWS, M.D., LL.D.
Professor J. BARTLETT, M.D.
Professor W. T. BELFIELD, M.D.
Professor F. BILLINGS, M.D.
Professor W. H. BYFORD, A.M., M.D.
Professor L. CURTIS, A.M., M.D.
Professor N. S. DAVIS, Jr., A.M., M.D.
Professor O. C. DE WOLF, A.M., M.D.
Professor C. FENGER, M.D.
Professor J. N. HYDE, A.M., M.D.
Professor E. F. INGALS, A.M., M.D.
Professor F. S. JOHNSON, A.M., M.D.
Professor H. A. JOHNSON, M.D., LL.D.
Professor C. W. PURDY, M.D.
Professor C. G. SMITH, A.M., M.D.
Professor E. WING, A.M., M.D.
Professor E. C. DUDLEY, A.B., M.D.

MILWAUKEE, WIS.:
Professor N. SENN, M.D., Ph.D.

WASHINGTON, D. C.:
S. O. RICHEY, M. D.

UNITED STATES ARMY:
Surgeon D. L. HUNTINGTON, M.D.

UNITED STATES NAVY:
Surgeon-General J. M. BROWNE, M.D. Medical-Director A. L. GIHON, A.M., M.D.

MARINE HOSPITAL SERVICE:
Surgeon S. T. ARMSTRONG, M.D., Ph.D.

